# From Gaze to Game: A Systematic Review of Eye-Tracking Applications in Basketball

**DOI:** 10.3390/brainsci15040421

**Published:** 2025-04-20

**Authors:** Michela Alemanno, Ilaria Di Pompeo, Martina Marcaccio, Daniele Canini, Giuseppe Curcio, Simone Migliore

**Affiliations:** 1 Department of Biotechnological and Applied Clinical Sciences, University of L’Aquila, 67100 L’Aquila, Italy; michela.alemanno@graduate.univaq.it (M.A.); ilaria.dipompeo@graduate.univaq.it (I.D.P.); martina.marcaccio@graduate.univaq.it (M.M.); simone.migliore@univaq.it (S.M.); 2 Department of Movement, Human and Health Sciences, University of Rome “Foro Italico”, 00135 Rome, Italy; d.canini@studenti.uniroma4.it

**Keywords:** eye movements, gaze behavior, visual search, sports, cognitive functions

## Abstract

**Background/Objectives:** Eye-tracking technology has gained increasing attention in sports science, as it provides valuable insights into visual attention, decision-making, and motor planning. This systematic review examines the application of eye-tracking technology in basketball, highlighting its role in analyzing cognitive and perceptual strategies in players, referees, and coaches. **Methods:** A systematic search was conducted following PRISMA guidelines. Studies published up until December 2024 were retrieved from PubMed and Web of Science using keywords related to basketball, eye tracking, and visual search. The inclusion criteria focused on studies using eye-tracking technology to assess athletes, referees, and coaches. A total of 1706 articles were screened, of which 19 met the eligibility criteria. **Results:** Eye-tracking studies have shown that expert basketball players exhibit longer quiet eye (QE) durations and more efficient gaze behaviors compared to novices. In high-pressure situations, skilled players maintain more stable QE characteristics, leading to better shot accuracy. Referees rely on efficient gaze strategies to make split-second decisions, although less experienced referees tend to neglect key visual cues. In coaching, eye-tracking studies suggest that guided gaze techniques improve tactical understanding in novice players but have limited effects on experienced athletes. **Conclusions:** Eye tracking is a powerful tool for studying cognitive and behavioral functioning in basketball, offering valuable insights for performance enhancement and training strategies. Future research should explore real-game settings using mobile eye trackers and integrate artificial intelligence to further refine gaze-based training methods.

## 1. Introduction

Eye tracking is a technique that measures the point of gaze or the motion of an eye relative to the head, providing insights into visual attention and cognitive processes [1]. By capturing data on where and how long individuals look at specific stimuli, researchers can infer underlying mental states and decision-making processes [2]. This technology has been instrumental in fields such as psychology, neuroscience, marketing, and human–computer interaction, offering a window into the intricate relationship between visual attention and behavior [3].

Recent advancements have made eye tracking more accessible and affordable, broadening its research applications. Innovations in machine learning and computer vision have improved the accuracy of eye-tracking systems, enabling more detailed analyses of visual behavior. These developments have opened new avenues for exploring the complex dynamics of attention and perception in real-world settings [4].

In psychology and neuroscience, eye tracking is used extensively to study cognitive processes such as attention, perception, and memory [1]. Moreover, it provides real-time data on how individuals process visual information, aiding in the understanding of various psychological phenomena [5].

Eye tracking has emerged as a pivotal technology in sports science, offering researchers and practitioners a window into the visual and cognitive processes that underpin athletic performance. By precisely recording where and for how long an athlete fixates on specific visual cues during training or competition, eye tracking enables a detailed analysis of perceptual strategies, decision-making, and motor planning. This technology has been applied across various sports—from basketball and soccer to tennis and golf—helping elucidate the mechanisms that differentiate expert performers from novices [6].

**Figure 1:**
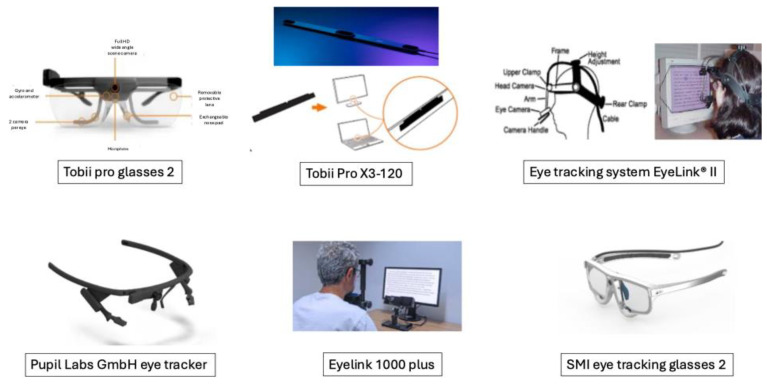
Some examples of mobile and fixed eye-tracking devices.

Modern eye-tracking systems range from head-mounted mobile devices to remote, screen-based systems (see Figure 1 for some examples). Originally developed for psychological research, eye tracking has evolved considerably with advances in sensor technology and computer vision. Modern mobile eye trackers, for instance, allow for the unobtrusive, in situ analysis of players during live games or training sessions. This technological progress has enhanced the ecological validity of studies, enabling researchers to capture data in realistic environments rather than in laboratory settings. These tools measure key metrics such as fixation duration, saccadic movements, and gaze dispersion, which are critical in understanding how athletes allocate their visual attention in dynamic and complex environments [2]. The advancement of sensor technology and computer vision has led to more unobtrusive and accurate devices that can capture real-world performance data. This progression has enhanced the ecological validity of studies, allowing research to move from controlled laboratory settings to the authentic contexts of competitive sport.

Eye tracking has proven invaluable across several key areas. In decision-making, analyzing where athletes direct their gaze during game situations helps researchers understand how they process spatial and temporal information—an essential component for making split-second decisions. Additionally, eye tracking enhances training and feedback; coaches can use the data to offer targeted advice, such as refining gaze strategies to improve shot selection in basketball or to enable more efficient field scanning in soccer. Comparative studies also reveal distinct differences in gaze patterns between expert and novice athletes, with experts typically exhibiting more efficient visual search strategies that correlate with better anticipation and performance in high-pressure situations.

A key limitation of the existing research lies in the ecological validity of experimental paradigms. Many eye-tracking studies in sport have relied on static or semi-dynamic stimuli (e.g., video-based decision-making tasks) that fail to replicate the temporal and spatial complexity of in situ performance. Such approaches may overlook how perceptual–motor coupling unfolds during live play, where athletes must not only extract critical information but also continuously integrate it with motor execution under time pressure. Additionally, there is insufficient exploration of individual differences in gaze behavior across player positions, tactical roles, and developmental stages. This leaves open questions regarding the adaptability and transferability of perceptual skills across varying task demands. Methodologically, moreover, the field continues to grapple with several challenges. Mobile eye-tracking systems, though increasingly advanced, are still constrained by issues of data loss due to head movement, occlusion, and limited sampling rates in fast-paced sports. Synchronizing gaze data with contextual game metrics (e.g., player positioning, ball trajectory) requires sophisticated multi-modal data integration that is both technically and analytically demanding. Furthermore, establishing causal links between gaze behavior and performance outcomes necessitates designs that go beyond correlational evidence to include manipulations of attentional focus and training interventions.

The aim of this review is to explore the current state of knowledge regarding the application of eye-tracking technology in basketball.

## 2. Materials and Methods

The current systematic review was carried out based on the guidelines and principles outlined by the Preferred Reporting Items for Systematic Reviews and Meta-Analyses (PRISMA) statement 2020 and checklist [7].

### 2.1. Search Strategy and Study Selection

We searched the PubMed and Web of Science databases for studies published between January 2004 and December 2024, as technology prior to this period may be difficult to compare with more recently advanced technology. This research utilized a search string containing the following keywords: (basket OR basketball) AND (eye-tracker OR eye tracker OR eye tracking OR eye-tracking OR visual search OR gaze OR fixation OR vision OR quiet eye OR visual).

### 2.2. Inclusion and Exclusion Criteria

We included studies about basketball using eye-tracking devices. In the first step (identification), we excluded reviews, systematic reviews, meta-analyses, and animal studies from the final database. In the second step (screening), we excluded studies in which there were no basketball themes and research without eye tracker support. In the end (included), we included articles that studied basketball from different points of view using eye trackers. We included studies on basketball players, referees, and coaches regarding how they analyze the match or single actions like free throws, passing the ball, making a shot, etc.

### 2.3. Data Extraction and Analysis

The PRISMA recommendations for systematic literature analysis were strictly followed. Studies were independently selected by two different authors, who first analyzed the titles and abstracts and subsequently selected the full papers meeting the inclusion criteria, resolving disagreements through consensus.

## 3. Results

From 1706 studies found at the beginning, 19 articles have been included in this review.

**Figure 2:**
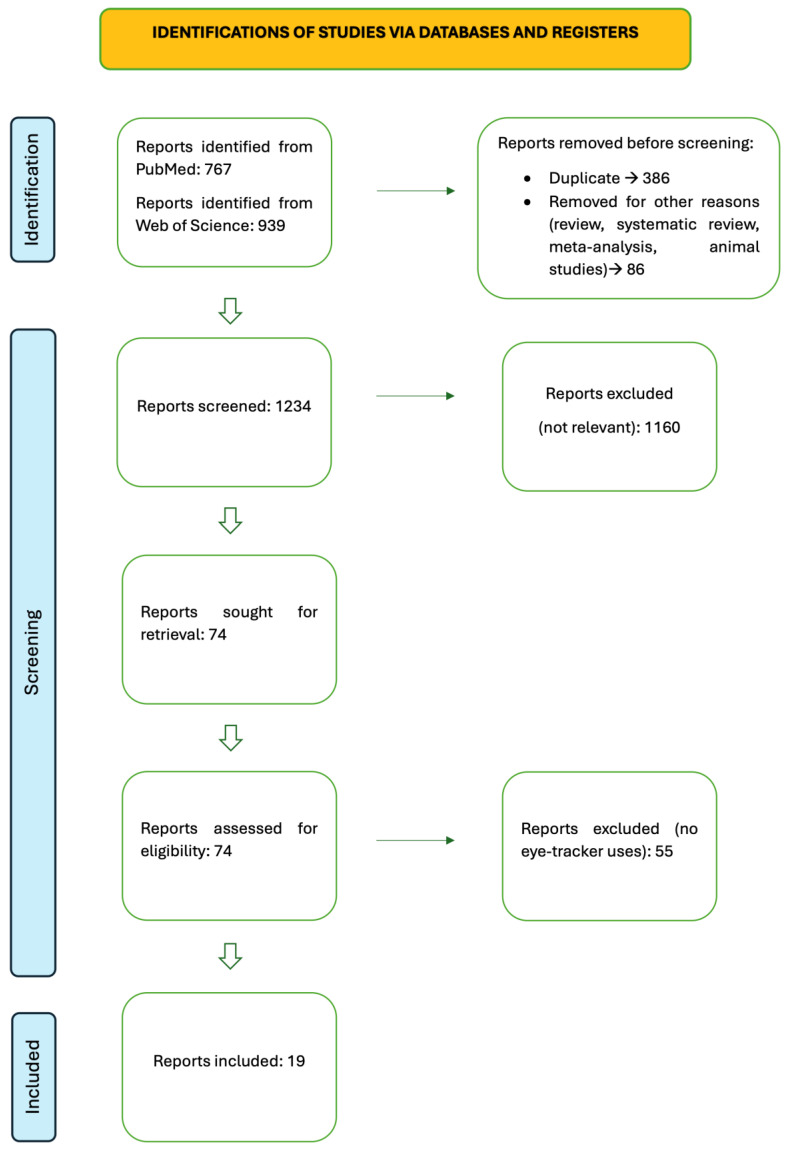
Preferred Reporting Items for Systematic Reviews and Meta-Analyses (PRISMA) diagram demonstrating search strategy.

The literature analysis and selection is summarized in a PRISMA-like diagram (see Figure 2). Each study has been analyzed, and a summary is reported in Table 1. In organizing the table, we considered the following: the participants, the cognitive domain assessed with regard to executive functions, the types of measures and materials used, methodological comments, and relevant results.

### 3.1. Literature Search

At the beginning, we selected 1706 articles. First, we removed the duplicates and all the reviews, systematic reviews, and meta-analyses. We had at that point 1234 articles. Then, we excluded all the articles that were not relevant, and we selected 74 studies. At the end, we again excluded the studies in which there was no eye tracking employed, and we started our study with 19 articles.

### 3.2. Participants’ Characteristics

In this review, we wanted to evaluate behavior and cognitive functioning related to basketball through the use of eye-tracking technology.

We found fourteen studies examining basketball players’ points of view, two studies on referees’ perspectives, and three studies on coaches’ perspectives. In the section dedicated to basketball players, we categorize the studies based on the game situation examined (free throws, three-point shots, and jump shots). A separate paragraph is dedicated to studies analyzing specific cognitive functions in basketball players through computerized tasks. The last two sections focus on studies describing referees and coaches in various game situations.

### 3.3. Athletes

#### 3.3.1. Free Throws

The free throw is a closed motor skill, performed under stable and predictable conditions, allowing for refined technique through repetition. The movement is a coordinated sequence beginning with lower-body force generation (hip and knee extension), transitioning through the trunk and shoulder, and culminating in elbow extension and wrist flexion (the “follow-through”). Free throws are sensitive to psychological stress; routine consistency (e.g., dribble count, breathing techniques) reduces cognitive load and improves accuracy.

One study [12] wanted to investigate the visual tracking strategies of expert and amateur basketball players during free-throw shooting. Specifically, it aimed to (1) compare the accuracy of free throws between 11 expert and 11 amateur players; (2) analyze the duration of the “quiet eye” (QE) during accurate and inaccurate free throws; (3) determine whether expert players exhibit longer QE durations and better visual strategies than amateur players; and (4) explore how these visual strategies can be taught to amateur players to enhance their performance. The QE was introduced by Vickers [27,28], and it refers to the final fixation on a target, such as the hoop, immediately before a player initiates a shot. The study found that expert players had higher accuracy in free throws compared to amateur players. During accurate throws, expert players had a longer QE duration compared to amateur players. For inaccurate throws, the durations were again longer for experts and shorter for amateurs. Expert players spent more time fixating on the hoop compared to amateurs during accurate throws. For inaccurate throws, experts had a fixation time longer than that of amateurs. The findings suggest that expert players have longer QE durations and better visual strategies, which contribute to their higher accuracy in free throws.

Another study [11] tried to investigate the eye movement characteristics exhibited by 20 female basketball players during free throws at varying exercise intensities and to explore the relationship between these eye movement characteristics and the free-throw percentage. The study aimed to understand how different levels of exercise intensity affect players’ visual focus and how these changes correlate with their free-throw success rates. They found that the average number of fixations on the hoop and net showed significant differences across different exercise intensities: high-intensity free throws required more fixations compared to low and moderate intensities. The fixation durations on the hoop, backboard, and net also exhibited significant differences: the hoop had the highest proportion of overall fixation duration, with longer durations needed for high-intensity free throws. For moderate-intensity free throws, there was a significant negative correlation between the number of fixations on the hoop and the free-throw percentage. For high-intensity free throws, there was a significant positive correlation between the fixation duration on the hoop and the free-throw percentage. These results suggest that exercise intensity affects players’ visual focus and processing, impacting their free-throw success rates.

An interesting study [9] investigated the relationship between an “especial skill” and the QE duration in basketball free throws. The “especial skill” mentioned in the study refers to a phenomenon observed in basketball free throws. It describes how players perform significantly better at the free-throw line than would be predicted based on their performance from other nearby distances. This effect is attributed to the extensive practice and repetition of free throws, which creates a highly specific and dense sub-space of task solutions within the general skill of set shots. This specialized practice leads to higher accuracy and longer QE durations at the free-throw distance compared to other distances. Specifically, the study aimed to test the inhibition hypothesis, which suggests that longer QE durations in expert athletes are due to increased inhibition requirements over movement parametrization. The study examined whether prolonged QE durations are necessary to shield the optimal task solution against alternative solutions in the highly practiced skill of basketball free throws. The study found that the participants, one female and fifteen male basketball players, showed higher shooting accuracy from the free-throw line than predicted based on performance from nearby distances; the QE durations at the free-throw distance were significantly longer than predicted. These findings support the inhibition hypothesis, suggesting that prolonged QE durations are necessary to inhibit alternative task solutions and optimize performance in highly practiced skills like basketball free throws.

Another study [24] wanted to determine how two types of practice (constant and variable) affect gaze behavior in basketball free throws. The researchers hypothesized that different practice conditions might lead to different gaze behaviors, which could explain why variable practice gives an advantage in novel situations and constant practice in trained conditions. The study also aimed to explore whether constant practice would result in the development of an “particular skill”, which is a variation of a skill that is significantly better performed compared to other variations, even if all variations are practiced equally. This phenomenon is observed when a specific variation of a skill, such as a basketball free throw from a standard distance, is practiced extensively in constant conditions, leading to superior performance at that specific distance compared to other distances. Twenty males without experience in basketball took part in the study. In the experiment, there were two conditions: the pre-test and post-test. The study found that the total fixation duration on the basket rim significantly increased in the post-test compared to in the pre-test, regardless of practice conditions (constant or variable); the number of fixations also increased significantly in the post-test; the average fixation duration increased in both groups, but the increase was not statistically significant; there were no significant differences in the gaze behavior between the constant and variable practice groups; the study did not find evidence of the development of an “especial skill” in the constant practice group. These results suggest that both constant and variable practice conditions lead to similar improvements in gaze behavior, but neither condition resulted in the development of a highly refined skill variation.

With regard to practice and training, another study [26] tried to evaluate an augmented reality (AR)-based training system for basketball free throws. The system projects the optimal shot trajectory using a head-mounted display (HMD) based on the shooter’s release point. The study aimed to assess the efficacy of this AR training system by comparing changes in success rates and eye-gaze behavior (QE duration) between novice shooters using AR training and novice shooters using conventional training methods. Twenty novice basketball players took part in the experiment, 10 in the AR training group and 10 in the conventional training methods group. The goal was to determine if the AR system can improve free-throw shooting performance and visual attention in novice basketball players. The study found that the AR training system for basketball free throws had some interesting results: the success rate of free throws increased significantly in the post-training phase for the AR group. In contrast, the control group showed no significant change in the success rate; the AR group exhibited a longer QE duration during the AR training phase compared to the pre- and post-training phases. The control group showed no change in the QE duration across all phases; the AR training system improved free-throw performance and visual attention (QE duration) in novice shooters, suggesting that the system can enhance basketball free-throw shooting performance. These results indicate that the AR-based training system positively impacted both the shooting performance and visual attention of novice basketball players.

#### 3.3.2. Three-Point Shots

Three-point shooting in basketball is a dynamic and technically demanding skill that integrates biomechanical precision, neuromuscular coordination, perceptual awareness, and projectile mechanics. Unlike free throws, which are performed from a fixed position, three-point attempts are often executed under variable conditions—on the move, off the dribble, or under defensive pressure. From a biomechanical perspective, the three-point shot involves a coordinated kinetic chain beginning from the lower body and extending through the upper limbs. To generate sufficient force for the increased distance, shooters rely on enhanced knee and hip flexion during the preparatory phase. The shooting action itself requires precise control of elbow extension and wrist flexion. Beyond physical mechanics, three-point shooting also places significant demands on the athlete’s perceptual and cognitive systems.

The objective of one study [13] was to understand the role of QE during basketball three-point shots, particularly under high-pressure game conditions. The researchers aimed to examine the impact of time and performance pressure on QE characteristics and shot accuracy and investigate how QE contributes to attentional control and performance, especially in critical game moments, comparing the QE behavior between twelve competitive elite and nine semi-elite basketball players. The results showed that competitive elite players had a longer QE online duration and a shorter QE preprogramming duration compared to semi-elite players, especially under high-pressure conditions; time and performance pressure significantly affected the QE characteristics. Semi-elite players showed more unstable QE under pressure, while competitive elites maintained more stable QE characteristics. Despite the pressure, competitive elites maintained higher shooting accuracy than semi-elites, indicating their ability to use QE effectively to cope with high-pressure situations. These findings suggest that QE plays a crucial role in attentional control and performance, particularly for elite players in high-pressure game scenarios.

Connecting to the previous study, another research study [22] wanted to investigate the efficacy of QE training in improving the accuracy of basketball three-point shots under pressure. It aimed to determine whether QE training can help players maintain their visual attention and performance in under-pressure situations. The study involved 18 expert male basketball players and examined their gaze behaviors and shooting accuracy through various tests. The study found that QE training significantly improved the performance and accuracy of basketball three-point shots under pressure: the QE-trained group showed significantly better accuracy in the pressure test compared to the control group.

#### 3.3.3. Jump Shots

A jump shot in basketball is a basic shooting technique where a player jumps, holds the ball, and releases it at the peak of their jump, aiming for the basket. It is a fundamental move, often used to take shots from various positions on the court, including from a standing position or while moving, like during a drive to the basket.

The objective of one study [20] was to examine the visual patterns in 10 male novice youth and 10 professional adult basketball players while performing a jump shot. The study aimed to identify differences in visual strategies, such as the QE time, number of fixations, and fixation durations, between these two groups. The authors found that youth players had lower shooting accuracy compared to professional players at both long and middle distances; professional players had longer QE times than youth players at both long and middle distances; professional players had longer total fixation durations at both long and middle distances; and youth players had a greater number of fixations compared to professional players at long distances. These results suggest that professional players use more efficient visual strategies, which could be beneficial for training youth players to improve their shooting performance.

#### 3.3.4. Shot Deception

In basketball, shot deception is a deceptive move where an offensive player simulates a shot attempt to trick their defender. The goal is to make the defender react prematurely, creating opportunities for the offensive player to shoot, drive, or pass.

Another study [16] wanted to examine the sources of deceptive information during the anticipation of shot fakes in basketball. The study aimed to investigate the effects of shot deception on players’ anticipation behavior and to compare the gaze behavior and anticipation performance of 15 expert and 16 novice basketball players when defending against genuine and faked basketball shots. In particular, they wanted to identify the body regions that convey genuine and deceptive shot information, focusing on how gaze fixations on different body parts (e.g., hips, legs, ball, head) influence anticipation accuracy. It emerged that experts had higher accuracy in anticipating shot fakes compared to novices, especially for ball fakes. Concerning the gaze behavior, more fixations on the hips and legs were associated with successful anticipation, and more fixations on the ball and head were linked to unsuccessful anticipation. These findings suggest that focusing on the hips and legs can improve anticipation accuracy and that experts use more effective gaze strategies to counteract deception in basketball.

#### 3.3.5. One-on-One Defenders

In basketball, one-on-one defense refers to a defensive situation where a single defender is responsible for guarding one offensive player. The defender’s main goal is to prevent the offensive player from scoring by staying in front of them, disrupting their moves, and limiting their options.

One study [17] aimed to examine the use of defensive gaze strategies by defensive players in basketball by evaluating the on-field gaze behavior of basketball players defending in one-on-one situations, comparing the gaze behavior of expert and novice players. The study involved interviews with four national-level expert basketball coaches and a field study using mobile eye-tracking devices on sixteen expert and sixteen novice players to assess the alignment between coaching instructions and the gaze behavior of players. A discrepancy between the coaches’ advice and the players’ behavior emerged: coaches recommended focusing on the torso to avoid fakes; expert players mainly fixated on the head during the receiving and dribbling phases and on the ball during the shooting phase. There was also a difference between expert and novice players: experts primarily fixated on the head during the receiving and dribbling phases; novices focused more on the ball across all phases. Fixating on the ball or head potentially leaves defenders vulnerable to deceptive movements, as shown by video-based research. These findings highlight the need for more research on defensive gaze strategies and their practical implications for coaching.

#### 3.3.6. Cognitive Functioning and Visual Search Behaviors

One study [23] wanted to compare the saccadic eye movement ability of eight female professional basketball players with that of eight non-athletes. The researchers hypothesized that professional basketball players would exhibit more accurate saccadic eye movements compared to non-athletes. During the experiment, the subjects were tracking a visual target that moved continuously in a regular triangular wave-like pattern horizontally for 20 s. The visual target moved at a speed of 100 degrees per second, and the subjects were instructed to track the visual targets with maximum accuracy. They found that female professional basketball players exhibited significantly more accurate and consistent saccadic eye movements compared to non-athletes. These findings suggest that professional basketball players may use predictive saccades to track moving targets more accurately, even outside of actual competition.

Another study [19] tried to examine the differences in the performance of visual search tasks among 42 basketball players of different skill levels, considering the influence of different object working memory loads. The study aimed to explore the impact of object working memory load on the eye movement processes involved in visual search tasks among basketball players, analyze cognitive differences in the visual search between basketball players of different skill levels, and differentiate elite athletes from novices based on their visual search performance under varying working memory loads. The study found that object working memory load significantly impacts the performance of visual search tasks among basketball players. The accuracy of the visual search decreased with the inclusion of object working memory load; reaction times increased significantly with object working memory load; the number of gaze fixation points increased, and gaze trajectories became more complex under object working memory load; competitive elite athletes had shorter reaction times and fewer gaze fixation points compared to semi-elite and novice players, indicating superior visual search abilities. These findings suggest that higher working memory loads negatively affect visual search performance, with elite athletes showing better resilience to these effects.

Another study [18] examined the visual search strategies of 48 skilled basketball players during an anticipation task. Specifically, it aimed to compare visual search behaviors between experienced and inexperienced basketball players, analyzing the response time, accuracy, and eye movements during decision-making tasks involving offensive patterns in basketball and investigating the differences in fixation counts and durations on key areas of interest between expert and novice players. They wanted to understand how visual search strategies contribute to better anticipation and decision-making in basketball. They found that expert players had faster response times and higher accuracy in predicting offensive plays compared to novices; expert players spent a greater percentage of fixation duration and counts on relevant areas and less on irrelevant areas than novices. They also demonstrated more concise fixation trajectories, focusing mainly on key information areas, while novices had scattered and irregular fixation points. These results indicate that experienced basketball players employ more efficient and effective visual search strategies, leading to better anticipation and decision-making in game situations.

Finally, one study [8] wanted to investigate the role of top–down and bottom–up processes during a sports strategy called “no-look passes” and how microsaccades and small saccades modulate these processes. They divided the 24 subjects into 12 expert and 12 amateur basketball players, and they led two experiments. The first experiment examined the role of expertise in modulating the shift of covert attention with the bottom–up procedure; the second experiment investigated the shift of covert attention between the top–down and bottom–up conditions in a group of expert basketball players.

The top–down process is driven by higher-level cognitive functions such as goals, prior knowledge, and expectations. Expert basketball players use their knowledge and experience to decide where to pass the ball without external cues. The bottom–up process is driven by external stimuli that capture attention. Players respond to a visual signal like a teammate that raises their hand to call for the ball. The findings aimed to provide insights into how athletes use visual attention and eye movements to perform deceptive strategies in sports. In experiment 1, it emerged that amateurs exhibited more saccades with greater amplitude and faster peak velocity compared to experts; there were no significant differences in the microsaccade characteristics (rate, amplitude, peak velocity, and duration) between amateurs and experts. In experiment 2, they found that during the bottom–up condition, athletes exhibited more microsaccades due to peripheral stimulation. In the top–down condition, athletes exhibited more small saccades to decide where to send the ball. Athletes were faster in making passes during the top–down condition compared to the bottom–up condition. These results suggest that expertise influences eye movement patterns, with experts showing more stability during fixation. The type of attentional process (top–down or bottom–up) affects the frequency and type of eye movements made during a deceptive sports strategy like no-look passes.

### 3.4. Referees

One study [10] aimed to analyze how basketball referees’ gaze behavior and stimulus selection vary based on visual angle perspective, comparing lead and trail positions, as well as the level of expertise, comparing expert and novice referees. The study examined 16 basketball referees, and the researchers found that referees in the lead position focused more on the attacking player with the ball, while those in the trail position had a broader visual focus; expert referees showed more efficient gaze patterns, focusing on critical areas more quickly and accurately than novices; referees used a visual pivot on the players’ trunk to maintain awareness of the game dynamics. These findings suggest that both the position on the court and the level of expertise significantly influence how referees visually process the game.

Another study [25] aimed to evaluate the gaze behavior and positioning of nine high-class basketball referees during three-point shots. Specifically, it aimed to assess the extent to which referees fulfill their assigned tasks and responsibilities during three-point shots and determine the effectiveness of referees’ gaze allocation and how it aligns with International Basketball Federation (FIBA) recommendations. Furthermore, the researchers wanted to investigate the overlap in gaze behavior among referee teams to understand their coordination and decision-making processes. They found that referees positioned near the ball covered their primary areas of responsibility more effectively than those farther away. Lead referees focused more on players inside the shot zone and less on the basket. Center and trail referees near the ball looked at the basket more often, aligning with FIBA guidelines. Referees farther from the ball tended to watch the shooter more, potentially neglecting other responsibilities. Referees rarely directed their gaze at the same area simultaneously, indicating the effective distribution of visual attention. Gaze behavior varied across the three phases of the shot, with referees adjusting their focus from the shooter to the basket and players fighting for rebounds. These findings suggest that referees farther from the ball may need to improve their focus on non-ball-related actions to enhance decision-making accuracy.

### 3.5. Coaches

The objective of one study [14] was to investigate how coaches’ pointing gestures affect the allocation of players’ visual attentional resources and their memorization of tactical information in basketball. The study examined the differences in the effectiveness of these gestures between 48 expert and 48 novice players. The study found that coaches’ pointing gestures significantly improved the performance of novice basketball players but had no effect on expert players. It emerged that novices had better recall accuracy when coaches used pointing gestures; they invested less mental effort in the with-gesture condition. Novices showed more efficient visual search patterns with pointing gestures. Experts performed similarly in both the with-gesture and no-gesture conditions; their mental effort remained the same regardless of the condition. The experts’ visual attention patterns did not change with the use of pointing gestures. These findings suggest that the effectiveness of coaches’ pointing gestures depends on the players’ level of expertise.

Another study [15] wanted to investigate whether a coach’s gaze guidance can enhance the memorization of tactical movements in basketball. The term “gaze guidance” refers to the coach’s use of eye movements to direct the players’ attention during instructional sessions. It involves the coach shifting their gaze between the players and the relevant elements on a board or diagram. This method is used to redirect the players’ attention to specific areas of information, enhancing their focus and memorization of tactical movements in basketball. Specifically, the study examined how different gaze behaviors (direct gaze, guided gaze, and fixed gaze) affect players’ visual attention and recall performance, considering their level of expertise (72 novice vs. 72 expert players). The study found that the coach’s guided gaze significantly improved the recall performance and reduced the mental effort of novice basketball players. Novice players showed higher recall accuracy and lower mental effort compared to direct and fixed gaze conditions; they focused more on relevant diagrams and less on the coach’s face. On the other hand, for the expert players, recall performance and mental effort were similar across all gaze conditions. These results suggest that guided gaze is particularly beneficial for novices, helping them focus on key elements and improve their learning outcomes.

Finally, the last study [21] examined the visual search strategies of eight basketball coaches with different performance levels (four top level and four bottom level). The study aimed to identify patterns in how coaches visually explore the game during a normal basketball practice situation. They tried to understand how coaches’ visual search sequences vary depending on their performance level. The study found that top coaches prefer to start their visual search sequences with the interpersonal space category, using a variety of categories, including attacker to the side of the ball, defender to the side of the ball, and attacker with the ball. There were recurrences in their visual search patterns, indicating more consistent and repeated sequences. Bottom coaches often begin their visual search sequences with the attacker with the ball category, tending to focus more on the attacker to the side of the ball in their sequences. Top coaches exhibit more complex and varied visual search strategies, while bottom coaches tend to have more repetitive patterns. These differences highlight that top coaches have more systematic and varied visual search patterns, which may contribute to their effectiveness in coaching.

## 4. Discussion

This systematic review underscores the great contribution of eye-tracking technology to the understanding of the cognitive and behavioral processes underlying performance in basketball. Eye tracking is a powerful tool for investigating how athletes, coaches, and referees visually process and interpret different game situations. Eye tracking provides objective, real-time data on gaze patterns, fixation durations, and saccadic movements. This allows researchers to directly infer cognitive processes such as attention allocation, decision-making, and anticipation, offering insights that would be difficult to obtain otherwise.

The ability to precisely quantify visual behavior makes eye tracking uniquely suited to examining the cognitive differences that distinguish expert performers from novices. The studies reviewed demonstrate the utility of eye tracking in identifying key differences in visual strategies between individuals of varying skill levels. Whether comparing youth players to professionals in jump shots [20], expert versus semi-elite players in three-point shots under pressure [13], or expert versus novice referees [10], eye tracking reveals distinct patterns of visual attention that correlate with performance outcomes. For example, the finding that expert players often exhibit longer QE durations and more efficient visual search strategies highlights the importance of attentional control to have better results. Moreover, eye tracking is crucial in assessing training interventions and providing targeted feedback. The AR-based training system shows improvements in the free-throw success rate and QE duration in novice shooters [26], demonstrating the potential of eye tracking to refine gaze strategies and optimize athletic performance.

In the coaching domain, eye tracking has shown that pointing gestures and guided gaze are significantly beneficial for novice players but have less effect on expert players [14,15]. This suggests that the role of the coach shifts from direct instruction to aspects of individual management, group cohesion, and motivation as athletes become more skilled.

Concerning the referees, the referees’ gaze behavior varied based on their court position and expertise. Expert referees exhibited more efficient gaze patterns, focusing on critical areas more quickly and accurately. However, referees farther from the ball often neglected non-ball-related actions, highlighting a need for improved visual attention distribution [10,25].

These results share certain points of contact with those observed in other sports that have explored similar perceptual–cognitive mechanisms. For example, in basketball free throws, experts exhibit longer QE durations than novices, correlating with higher shot accuracy and better attentional control, especially under pressure [9,13,22,28]. These findings are consistent with golf putting [29] and archery [30], two sports that also involve self-paced, closed motor skills. Moreover, some basketball studies [13,22] show that elite players maintain stable QE and shooting performance under pressure, whereas semi-elites exhibit gaze disruption. Similar patterns emerge in soccer penalty kicks and pistol shooting, as shown by Wilson and colleagues [31] and Janelle and collaborators [32], respectively. Studies [18,19] on basketball showed that elite players engage in more efficient visual search patterns, with fewer, longer fixations on task-relevant cues. Similar results were seen in other fast-paced sports, for example in volleyball, where experienced setters were shown to fixate on the opponents’ shoulders and trunk to predict attack direction, minimizing fixations on irrelevant cues [33].

The studies in our systematic review and these cross-sport consistencies offer compelling support for several theoretical frameworks in cognitive neuroscience, notably the neural efficiency hypothesis, attentional control theory, and the inhibition hypothesis. The consistent observation that expert athletes exhibit prolonged QE durations prior to successful free throws (e.g., [9,12]) aligns with the neural efficiency hypothesis, which posits that expert performers require fewer cognitive resources for task execution due to optimized neural functioning [34,35]. The longer QE durations observed in experts suggest a more efficient allocation of attentional resources, allowing for superior visuomotor integration and response preparation [29].

Moreover, attentional control theory [36,37] is pertinent in interpreting findings where expert players maintained stable QE patterns under pressure, while less skilled players exhibited deteriorated gaze behavior and performance [13]. This indicates that expert athletes possess enhanced top–down attentional control mechanisms that buffer against cognitive interference, preserving both gaze stability and motor execution.

The inhibition hypothesis, advanced in sports science by researchers such as Klostermann et al. [38,39], finds empirical support in studies demonstrating that the prolonged QE in expert shooters is not merely a marker of preparation but functions to suppress competing motor solutions [9]. This capacity for selective inhibition is crucial for executing highly practiced, task-specific motor routines—termed “especial skills”—with exceptional consistency and accuracy.

Additional evidence from studies employing augmented reality (AR) training underscores the role of perceptual learning and sensorimotor adaptation. Novice players trained with AR systems not only demonstrated improved shooting accuracy but also exhibited extended QE durations, reinforcing the plasticity of attentional strategies through enhanced external feedback [26].

Findings on gaze behavior across varying exercise intensities further contribute to understanding cognitive load theory [40]. High-intensity conditions induced more fixations and longer QE durations [11], which may reflect increased demands on working memory and executive attention to maintain performance under physiological stress.

Collectively, these studies converge to highlight the complex interplay between perceptual–cognitive expertise, attentional regulation, and neural efficiency in motor performance. They also advocate for training methodologies that foster top–down control and gaze stabilization—hallmarks of elite performance in dynamic sport environments. However, the current literature reveals a noticeable gap: a scarcity of eye-tracking studies conducted in live game situations. Most of the reviewed studies focused on controlled training scenarios or simulated game environments. While these settings offer valuable experimental control, they may not fully capture the complexity and unpredictability of real competitive matches. Future research should prioritize the use of mobile eye-tracking devices to investigate visual behavior in ecologically valid contexts for a more comprehensive understanding of how athletes, coaches, and referees perform under the pressures of live competition. Additionally, integrating eye-tracking technology with other physiological and cognitive measures could provide a more comprehensive understanding of the factors influencing basketball performance.

Looking ahead, the integration of eye-tracking technology with other emerging technologies could be promising. Virtual reality and AR can provide immersive training environments. When combined with machine learning and artificial intelligence for data analysis, eye-tracking data can be used to develop predictive models of performance, personalize training programs, and maybe even identify potential risks for injury. These advancements could revolutionize the way athletes are trained and coached. As technology continues to evolve, eye tracking could become an indispensable tool for unlocking the secrets of athletic expertise.

### Limitations

Our review shows some limitations. First, the heterogeneity in study designs, sample sizes, devices used, and methodologies limits the generalizability of the results. For instance, some studies focused on specific game situations (i.e., free throws), while others examined cognitive functions, making direct comparisons challenging. Second, the reliance on laboratory settings in some studies may reduce ecological validity, as real-game dynamics differ from controlled environments. Third, the small sample sizes in certain studies may limit the robustness of the findings. Finally, the absence of longitudinal studies makes it difficult to assess the long-term impact of eye-tracking-based training interventions on performance.

## 5. Conclusions

In conclusion, the findings highlight the critical role of visual attention and gaze behavior in enhancing performance, decision-making, and tactical understanding across different levels of expertise.

We recommend that future research tailor eye-tracking methodologies to specific basketball contexts. For example, stationary eye trackers may be suitable for controlled, skill-specific assessments, whereas mobile eye-tracking systems are better suited for in-game scenarios that demand real-time perception–action coupling. Combining eye-tracking data with performance metrics such as decision accuracy, reaction time, and physiological parameters (i.e., heart rate variability) may also yield a more holistic understanding of visual–motor performance.

From a practical standpoint, coaches and practitioners can apply these findings to optimize training environments. Drills that simulate game-like conditions—particularly under time constraints or with manipulated visual fields—can help develop more effective gaze strategies. Additionally, integrating video-based feedback to train visual search behaviors or using eye-tracking technology during skill acquisition phases may accelerate perceptual–cognitive development.

Eye-tracking technology has proven to be a powerful tool for understanding and enhancing visual attention and performance in basketball. However, addressing the current limitations and expanding the scope of the research will be essential for maximizing its potential in both academic and practical contexts.

## Figures and Tables

**Table 1:** All studies related to basketball and eye-tracking technology.

Reference	Participants	Cognitive Domains Assessed	Device	Type of Visual Analysis	Methodological Comments	Results
A. Piras (2024) [8]	24 male basketball players 12 expert players 12 amateur players	Visual search behavior	Eye-tracking system EyeLink^®^ II	Microsaccadic movements	Comparisons between groups	Movement time initiation showed no significant differences between groups or for passing direction. Analysis showed no significant differences in microsaccade characteristics for expertise or passing direction. Significant differences between groups for small saccade rate, amplitude, and peak velocity were found. Amateurs showed more saccades of greater amplitude and faster peak velocity than experts. Results showed no significant difference between groups/passing direction for microsaccade and small saccade orientations.
André Klostermann (2019) [9]	1 female and 15 male basketball players (M age = 23.3 years, SD age = 6.4 years)	Visual search behavior	Pupil Labs GmbH eye tracker	Quiet eye duration	Comparisons within group	With regard to the comparison of the predicted and the actual QE duration for the free-throw distance, participants’ actual QE durations were longer than the predicted QE durations. When comparing shooting accuracy as a function of the QE duration, participants were slightly more accurate in long- vs. short-QE-duration trials.
Antonio J Ruiz et al. (2023) [10]	16 basketball referees (1 F): 8 in non-professional group (all male; age = 27.5 ± 5.04 years) 8 in professional group (7 M; age = 22.8 ± 1.88 years)	Visual search behavior	ASL SE5000 Gaze Tracking System	Specific point of gaze on the scene	Comparisons between groups	No statistically significant differences were found between the expert and novice groups in the number of fixations. According to the spatial locations of play, all the referees showed a greater number of fixations and total fixation time on the attacking player with the ball. Novice referees showed longer fixation time on the defensive player of the ball than experts. Novice referees dedicated more fixations to the ball’s flight than experts.
Chunzhou Zhao et al. (2024) [11]	20 female basketball players (mean: 21.56; SD: 2.47 years)	Visual search behavior	Tobii Glasses 3 eye tracker	Number and duration of fixations	Comparisons within group	Moderate intensity free throws resulted in the fewest average fixations, while high-intensity free throws resulted in the most. The majority of fixations, regardless of intensity, were focused on the hoop. The moderate-intensity condition also had the shortest average total fixation duration, followed by the low-intensity condition, with the longest duration occurring during high-intensity free throws. Essentially, increased exercise intensity appears to be associated with more frequent and longer fixations, primarily on the hoop.
Ece Ayaz Kanat et al. (2021) [12]	22 basketball players 11 experts (20.71 ± 1.57) 11 amateurs (18.75 ± 1.06)	Visual search behavior	Eye-tracking device Tobii Pro Glasses 2	Quiet eye duration and average fixation time	Comparisons between groups	In this study, it emerged that the quiet eye duration of the accurate and the inaccurate throws of expert basketball players was longer than that of the amateurs. The average fixation time spent on the hoop for the accurate shots was longer in the expert group, while the average fixation time spent on the backboard was longer in the amateurs. For inaccurate throws, the results were the same.
Francesco Giancamilli et al. (2022) [13]	21 male basketball players 9 semi-elite (M = 13.78 years; SD = 1.56) 12 competitive elite (M = 16.92 years; SD = 1.78)	Visual search behavior	Eye-Tracking Glasses 2 (SMI ETG 2, SensoMotoric Instruments GmbH	Gaze behavior	Comparisons between groups	The large effect sizes of expertise on QE late components (i.e., QE offset and QE online duration) and the small effect sizes on QED and QE early components (i.e., QE onset and QE preprogramming duration) seem to suggest a relevant role of QE late components in maintaining goal-directed attention during a three-point shot. Competitive elites had a longer QE online duration than semi-elites, especially when time and performance pressure occurred.
Houssem Be Chikha et al. (2022) [14]	96 participants (48 male novices, age = 22.4 years, SD = 3.33; 48 male experts, age = 24.3 years, SD = 1.97) 48 participants in the no-gesture condition (24 experts and 24 novices) 48 participants in the with-gesture condition (24 experts and 24 novices)	Visual search behavior	Tobii Pro Glasses 2 eye tracker	Visual attention location	Comparisons between groups	Novice participants focused more on AOI1 compared to expert participants. For novices, the presence of gestures increased attention to AOI1 and reduced switching between AOI1 and AOI2 (coach). Expert participants showed no significant difference in attention to AOI1 or saccade count based on the presence or absence of gestures.
Houssem Ben Chikha et al. (2023) [15]	144 males 72 expert basketball players (M age = 25.15, SD = 1.9) 72 novice players (M age = 22.25 years, SD = 1.73)	Visual search behavior	Tobii Pro Glasses 2 eye tracker	Gaze behavior	Comparisons between groups	Novice players showed more efficient visual search, focusing on relevant diagrams and less on the coach. There were a higher number of fixations on the coach and more frequent saccades between the coach and diagrams. Expert players showed consistent visual search patterns across all conditions, with a focus on relevant diagrams and minimal attention given to the coach.
Johannes Meyer et al. (2022) [16]	31 adult participants (6 female, 25 male): 16 novices (M age = 24.24, SD = 2.57); 15 experts (M age = 21.67, SD = 3.33)	Visual search behavior	Pupil Core mobile eye-tracking system	Gaze behaviors	Comparisons between groups	The study investigated the effects of shot deception on players’ anticipation behavior in basketball. For the gaze behavior, successful anticipations involved more fixations on the hips and legs. Unsuccessful anticipations involved more fixations on the ball and head. Fixating on the hips and legs was the most effective strategy for distinguishing between genuine and fake shots. Experts were better at anticipating deceptive actions but were still susceptible to high shot fakes.
Johannes Meyer et al. (2022) [17]	16 expert basketball players (M age = 24.44) 16 novice basketball players (M age = 20.25)	Visual search behavior	Eye tracker from SensoMotoric Instruments	Gaze behaviors	Comparisons between groups	The study found that experts primarily fixated on the head during the receiving and dribbling phases and novices mainly focused on the ball throughout all phases. Coaches emphasized the importance of peripheral vision for defenders. These findings suggest a discrepancy between coaching instructions and actual gaze behavior.
Peng Jin (2023) [18]	48 male basketball players An expert group (n = 24, 12 guards and 12 forwards; M = 20.36 years; SD = 2.72) A novice group (n = 24, 11 guards and 13 forwards; M = 21.65 years; SD = 2.19)	Visual search behavior	Tobii Pro X3-120 eye tracker	Fixation, fixation duration, and fixation count	Comparisons between groups	Expert players and novice players spent more time fixated on the K-AOI (key area of interest). There were no differences between the two groups. The study found that expert players demonstrated a greater percentage of fixation duration in the R-AOI (related area of interest) than novice players. The results also show that the expert group had a greater percentage of fixation counts in the R-AOI than novice players.
Qing Nian (2023) [19]	42 subjects Competitive elite group: 11 subjects Semi-elite group: 15 subjects Novice group: 16 subjects	Visual search behavior	Eye tracker Eyelink 1000 plus	Visual search, total reaction time, search initiation time, scanning time, verification time, and number of gaze fixation points	Comparisons between groups	Competitive elite athletes demonstrate a visual search advantage over semi-elite and novice athletes. Novices exhibit complex and disorganized eye movements, while elite and semi-elite athletes show simpler, more direct visual search patterns. This indicates that higher-skilled athletes are better at strategy-based searching and integrating information. Novices have the highest number of gaze fixation points, followed by semi-elite and then competitive elite athletes, indicating a more efficient visual search strategy in higher-skilled players.
Rui Marques et al. (2023) [20]	20 male basketball players 10 under 16 10 over 16	Visual search behavior	SMI ETG 2W	QE times and fixation duration	Comparisons between groups	The QE time was longer, and the same occurred for the total fixation duration, among professional players. The last fixation duration was shorter in youth players compared to professional adult participants at both distances. Under-16 youth players had a greater number of fixations in the long but not the middle distance. Professional players evidenced fewer fixations and longer duration of final fixations compared with youth players.
Rui Sousa Damas et al. (2013) [21]	8 team coaches of under-18 male participants 4 top level 4 bottom level	Visual search behavior	Eye tracker system, instrument mobile eye 1.35	Gaze direction	Comparisons between groups	Top coaches exhibit more deterministic and varied visual search patterns, focusing on multiple points of interest. Preferentially, they use the interpersonal space category to begin their visual search sequences. They utilize a variety of categories, including the attacker to the side of the ball, the defender to the side of the ball, and the attacker with the ball. Bottom coaches tend to focus more on the attacker with the ball, showing less variation in their visual search sequences.
S. Moeinirad et al. (2022) [22]	18 expert male basketball players (M age = 20.0 ± 4.75 years)	Visual search behavior	SensoMotoric Instruments (SMI) eye-tracking glasses	Quiet eye duration	Comparisons within group	The QE-trained group showed improved performance accuracy in post-tests and pressure tests compared to pre-tests. This group also exhibited a longer total QE duration of hits in post-tests and pressure tests compared to the control group. The QE-trained group demonstrated longer early QE duration in post-tests and pressure tests relative to the control group. The control group showed no significant changes in the total and early QE duration across the tests.
Shunya Tatara et al. (2024) [23]	8 F professional basketball players, 24.3 ± 2.4 8 F non-professional	Visual search behavior	ORTe EYENAC	Eye movements	Comparisons between groups	Compared to the non-athlete subjects, basketball players executed more accurate and consistent eye movements in response to the regularly repeating movements of the visual target. In the basketball player group, the timing of saccades is more concentrated than in the non-athlete group.
Stanisław H. Czyz et al. (2019) [24]	20 males without experience in basketball 10 in constant group (M age = 21.80; SD = 0.79) 10 in variable group (M age = 22.70; SD = 1.42)	Visual search behavior	Eye-tracking system Dikabilis	Gaze behaviors	Comparisons between groups	An analysis of gaze behavior revealed that the total fixation duration significantly increased in the post-test compared to the pre-test. The CG and VG groups did not differ from one another in the pre- or post-tests. The number of fixations significantly increased in the post-test.
Stefanie Klatt et al. (2021) [25]	9 male basketball referees (M = 33.6 years, SD = 4.5 years)	Visual search behavior	Eye-tracking system Pupil Core binocular	Gaze behavior	Comparisons within group	In phase 1 and phase 2, referees looked more at the shooter when in a far-away position compared to a position near the ball, whereas this was not the case in phase 3. For the offense, the fixations significantly increased for the center role compared to the trail and lead referees. The referee/referees near the ball spent more time looking at the basket in phase 3 in comparison to the referees in a position farther away from the ball. When it came to the shooters, the fixation times of the referees decreased during the trajectory of the ball/shot.
Yuki Ueyama et al. (2024) [26]	20 participants (5 F; age range 20–23) 10 AR group 10 Ctr group	Visual search behavior	Eye tracker AR HMD	Gaze behaviors	Comparisons between groups	The success rate during the AR training with the optimal trajectory did not differ from the pre-training rate; post AR training, the success rate increased. AR training increased the QE duration compared with that recorded during the pre- and post-training blocks. The control group showed no change in the success rate or QED. These findings imply that the AR training system affected QE behavior and improved free-throwing shooting performance after training.
